# The neonatal tetrahydrobiopterin loading test in phenylketonuria: what is the predictive value?

**DOI:** 10.1186/s13023-016-0394-2

**Published:** 2016-01-29

**Authors:** Karen Anjema, Floris C. Hofstede, Annet M. Bosch, M. Estela Rubio–Gozalbo, Maaike C. de Vries, Carolien C.A. Boelen, Margreet van Rijn, Francjan J. van Spronsen

**Affiliations:** University of Groningen, University Medical Center Groningen, Beatrix Children’s Hospital, PO box 30.001, CA33, 9700 RB Groningen, The Netherlands; University Medical Center Utrecht, Wilhelmina Children’s Hospital, Utrecht, The Netherlands; Academic Medical Center, Emma Children’s Hospital, Amsterdam, The Netherlands; Maastricht University Medical Center, Maastricht, The Netherlands; Radboud University Nijmegen Medical Center, Nijmegen, The Netherlands; Leiden University Medical Center, Leiden, The Netherlands

**Keywords:** Phenylketonuria, PKU, Tetrahydrobiopterin, BH4, Neonate

## Abstract

**Background:**

It is unknown whether the neonatal tetrahydrobiopterin (BH4) loading test is adequate to diagnose long-term BH4 responsiveness in PKU. Therefore we compared the predictive value of the neonatal (test I) versus the 48-h BH4 loading test (test II) and long-term BH4 responsiveness.

**Methods:**

Data on test I (>1991, 20 mg/kg) at *T* = 8 (*n* = 85) and *T* = 24 (*n* = 5) were collected and compared with test II and long-term BH4 responsiveness at later age, with ≥30 % Phe decrease used as the cut-off.

**Results:**

The median (IQR) age at hospital diagnosis was 9 (7–11) days and the age at test II was 11.8 (6.6–13.7) years. The baseline Phe concentrations at test I were significantly higher compared to test II (1309 (834–1710) versus 514 (402–689) μmol/L, respectively, *P* = 0.000). 15/85 patients had a positive test I *T* = 8. All, except one patient who was not tested for long-term BH4 responsiveness, showed long-term BH4 responsiveness. In 20/70 patients with a negative test I *T* = 8, long-term BH4 responsiveness was confirmed. Of 5 patients with a test I *T* = 24, 1/5 was positive at both tests and showed long-term BH4 responsiveness, 2/5 had negative results at both tests and 2/5 showed a negative test I *T* = 24, but a positive test II with 1/2 showing long-term BH4 responsiveness.

**Conclusions:**

Both a positive neonatal 8- and 24-h BH4 loading test are predictive for long-term BH4 responsiveness. However, a negative test does not rule out long-term BH4 responsiveness. Other alternatives to test for BH4 responsiveness at neonatal age should be investigated.

## Background

Phenylketonuria (PKU, MIM 261600) is an autosomal recessive disorder caused by a deficiency of the enzyme phenylalanine-4-hydroxylase (PAH, EC 1.14.16.1), which is mainly active in the liver. PAH converts the essential amino acid phenylalanine (Phe) into tyrosine using the cofactor tetrahydrobiopterin (BH4). A deficiency of the enzyme leads to increased Phe and normal to decreased tyrosine concentrations in blood and tissues. Untreated, this results in progressive and irreversible neurological damage (among others mental retardation and epilepsy) [1, 2]. Early and continuous treatment by reducing blood Phe concentrations to a large extend prevents patients from mental disability. Adequate blood Phe concentrations can be achieved by means of a Phe restricted diet and/or the relatively new BH4 treatment that has an effect in approximately 20-50 % of PKU patients [3].

In most parts of the world, BH4 is prescribed to patients from four years of age onwards, because no large studies on safety of BH4 treatment in patients under the age of four years are available. Yet, also at this age treatment with BH4 is promising [4, 5]. If safety is proven, starting BH4 treatment at a young age can be beneficial for a healthy dietary pattern as some patients find it hard to learn to eat new foods after being restricted for many years [6, 7]. Also advantages of a diet containing more natural protein intake have been suggested [8, 9].

If BH4 treatment is started at an early age, accurate tests to distinguish patients that are or are not responsive are of great importance. In recent years, several methods have been developed to predict long-term BH4 responsiveness. The most used method is the BH4 loading test. Historically this test was performed at neonatal age, immediately after neonatal screening and aimed to distinguish hyperphenylalaninemic patients with PAH deficiency and patients with a BH4 deficiency [10, 11]. For this purpose an eight hour test is sufficient [12], although a 24 h test may be more effective in detecting dihydropteridine reductase deficiency.

In Europe, the standard test to evaluate BH4 responsiveness in PAH deficiency is a BH4 loading test taking at least 48 h and is for the greater part performed in patients of at least four years of age [13]. This elongation of the test seems to be important as it has been shown that a positive response to BH4 frequently occurs after the eight or even 24 h time point [14–17]. In the US even a 28-days method is used for all ages. Yet, these tests are inappropriate for the use in neonates as prolonged exposure to high Phe concentrations has a great impact on mental development at this age [18, 19]. BH4 loading tests postponing dietary treatment at neonatal age should be as short as possible to prevent unnecessary damage. This is extra important as the majority of the PAH deficient patients does not show BH4 responsiveness.

However, no study has investigated the predictive rate of the BH4 loading tests at neonatal age in comparison to later BH4 responsiveness. Therefore, our objective was to look at the discriminating ability of the historical neonatal BH4 loading test by comparing these tests to BH4 loading tests performed in the same patients at a later age.

## Methods

### Subjects

A historical cohort study was conducted comprising of patients with PKU and data available from both a neonatal BH4 loading test (test I) and a 48-h BH4 loading test (test II) at later age. Only patients born after 1991 who were tested with 20 mg/kg of BH4 during test I were included. The early diagnosed and treated patients were selected from six Dutch University Medical Centers and parts of the data have been reported before [17]. Patients with blood samples missing that hinder comparison of the tests were excluded from analysis. All data were collected from electronic and paper patient records. The medical ethical committee of the university medical center Groningen has ascertained that the protocol is not a clinical research with test subjects as meant in the Medical Research involving human subjects act (WMO). Therefore the local medical ethical committee had no task in reviewing the protocol.

### Neonatal BH4 loading test

The neonatal BH4 loading test (test I) was performed using 20 mg/kg BH4 (Schircks Laboratories), with 6R,S-BH4 until October 1999 and 6R-BH4 since October 1999. The BH4 was administered orally directly after taking a baseline blood sample. In most cases further blood samples were taken four and eight hours after BH4 administration and in some cases also after 24 h. The blood samples were collected as venous samples or with heel prick on filter paper, with the same sampling and analyzing method per patient. All patients were on a normal diet during the test.

### 48-h BH4 loading test

The 48-h BH4 loading test (test II) was conducted as described earlier [20], with 2 days of 20 mg/kg/day BH4 (Kuvan®) and filter paper blood samples collected on *T* = 0, 8, 16, 24 and 48 h. Patients with blood Phe concentrations below 400 μmol/L where supplemented with Phe (L-Phe in powder, a protein rich supplement such as milk powder or an increase in natural protein intake) until the test was finished. In both tests a reduction in Phe concentration of at least 30 % (during at least one sampling moment) was considered as positive. Patients with 30 % or more decrease of blood Phe concentration during test II were invited to investigate whether they also showed long-term BH4 responsiveness as described previously [17].

### Statistical analysis

Data were tested for normality using the Shapiro-Wilk test. Data with a normal distribution are presented as mean ± SD, whereas data with a skewed distribution are presented as medians with interquartile ranges (IQR). To compare continuous non-parametric data the Wilcoxon Signed Rank test was used. For comparing categorical data the Fisher’s exact test was used. The independent samples *t*-test corrected for unequal variances in case the Levene’s test was <0.05 was used for comparing parametric continuous data. All tests were performed two-tailed. The significance level was set at *p* < 0.05. Statistical analyses were performed using IBM Corp. SPSS Statistics for Windows, Version 20.0. Armonk, NY, USA.

## Results

In total, data on 88 early diagnosed patients were collected on both test I and test II. One patient was excluded because the *T* = 8 and *T* = 24 h Phe measurements were missing in test II. In 85 patients data included a *T* = 8 blood sample in both test I and test II. Three of these patients and two more patients (without *T* = 8 blood sample) were tested for 24 h during test I. Table 1 shows the demographic and clinical characteristics of the included patients. Although 60 % of patients received Phe supplementation during the test II, the baseline Phe concentrations were significantly lower compared to test I (*P* = 0.000).

**Table 1:** Demographic and clinical characteristics

	Total *n* = 87 patients
Sex	
Male	43 (49.4)
Female	44 (50.6)
Age at diagnosis (days)^a^	9 (7 – 11)
Phe at diagnosis (μmol/L)^b^	1319 (760 – 1820)
Phe at T = 0 1^st^ test (μmol/L)	1309 (834 – 1710)
Age at 2^nd^ test (years)	11.8 (6.6 – 13.7)
Phe at T = 0 2^nd^ test (μmol/L)	514 (402 – 689)

Data are n (%) or median (IQR).^a^ Not know in 2 patients. ^b^ at the first hospital visit (missing in 2 patients)

**Fig. 1:**
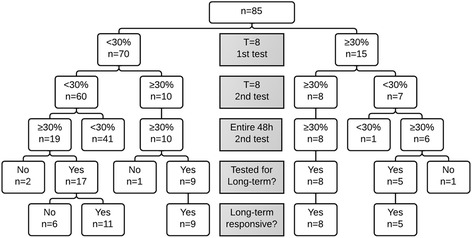
1st test, 2nd test and long-term BH4 treatment results in all patients with (at least) an 8-h neonatal BH4 loading test

Of the 85 patients with a *T* = 8 h Phe measurement during the test I, 70 showed less than 30 % decrease in Phe concentration. An overview of test I, test II and long-term BH4 responsiveness results of all patients with a *T* = 8 Phe measurement is shown in Fig. 1. Of 15 patients with 30 % or more Phe decrease at *T* = 8 during test I, fourteen had a positive test II, although not always within eight hours. All, except for one patient who did not continue BH4 treatment, showed long-term BH4 responsiveness. In 29/70 patients with less than 30 % Phe reduction at *T* = 8 during test I, 30 % or more Phe decrease at some moment during the entire test II was seen. Of these patients 26 were tested for their long-term response to BH4, which could be confirmed in twenty of them.

Of 5 patients with a *T* = 24 Phe measurement during the test I, two patients had the same negative result at *T* = 24 in test II and also less than 30 % Phe concentration reduction during the entire 48 h of test II. Two other patients had less than 30 % Phe concentration decrease in test I, but over 30 % in test II. Therefore both patients were tested for long-term BH4 responsiveness, during which one patient showed long-term BH4 responsiveness (6.5 g increase in natural protein with comparable Phe concentrations). One other patient had positive results at *T* = 24 in both tests and proved to show long-term BH4 responsiveness.

**Fig. 2:**
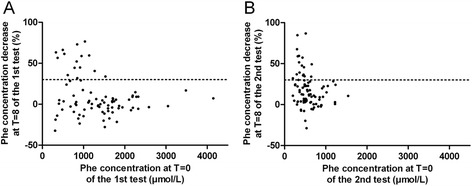
Scatter plot of the phenylalanine concentration at *T* = 0 and the percentage of Phe decrease at *T* = 8 of (**a**) the 1st test (**b**) the 2nd test

No significant difference was detected between the proportion of responders at T = 8 in test I and test II (15/85 and 18/85, respectively, *P* = 0.699). Patients with at least 30 % Phe reduction at *T* = 8 in test I had a mean baseline Phe concentration of 833 ± 333 μmol/L. This is significantly higher than the baseline Phe concentration of patients at least 30 % Phe reduction at *T* = 8 in test II (410 ± 119 μmol/L, P = 0.000). For both test I and test II, the baseline Phe concentration as well as the Phe reduction at *T* = 8 is shown for all patients in Fig. 2.

## Discussion

The present study shows that a neonatal 8-h test is inadequate in determining BH4 responsiveness, as at least 29 % of the patients with a negative 8-h neonatal BH4 loading test showed long-term BH4 responsiveness at later age. Even in the small group of patients tested for 24 h at neonatal age, long-term BH4 responsiveness, proven at later age, can be missed. However, the frequency of response in an 8-h test is not different between test I and II. Patients with a positive test I showed this response despite the significantly higher baseline Phe concentration compared to test II.

The first aim of neonatal screening always has been to start treatment as early as possible. This is very important as a delay in treatment has consequences for mental development. Treatment with BH4 could offer patients a diet with more or even a normal amount of natural protein. Consequently, it is desirable to know whether a patient is BH4 responsive or not at a young age. Historically, a short neonatal BH4 loading test was necessary for the distinction between PKU and BH4 deficiencies. Although a growing number of centers can discriminate between these deficiencies by measuring pterins and dihydropteridine reductase activity, the information gathered by the neonatal BH4 loading test is collected faster. Therefore, there still are a lot of centers for which the neonatal loading test is crucial. According to a study by Feillet et al., *n* = 9 PAH deficient patients with a 24-h neonatal BH4 loading test (20 mg/kg BH4) needed less time to reach Phe concentration under 300 μmol/L compared to *n* = 10 patients who started with a Phe restricted diet immediately after diagnosis [21]. This study nicely shows that neonatal use of BH4 in PAH deficient patients can effectively reduce the Phe concentration. Having said this, we need to stress that this does not take into account that the majority of patients is not responsive to BH4 [3]. More importantly, the patients that do not respond are often those with the highest Phe concentrations in which treatment is the most urgent [22].

One of the reasons explaining the lack of accurateness of the BH4 loading test at neonatal age in predicting long-term BH4 responsiveness is that at neonatal age Phe concentration is heavily influenced by the catabolic or anabolic state of the infant. Alternative methods to predict BH4 responsiveness could be the genotype, albeit that some inconsistent results exist [17, 23]. Another alternative could be to start BH4 treatment simultaneously with dietary Phe restriction and to stop administering BH4 when the metabolic control is stably within treatment ranges. A few studies have shown that in patients treated with BH4 Phe concentrations rise directly after missing a dose of BH4 or stopping the treatment [24, 25]. Therefore, a rise in Phe concentration is expected when BH4 treatment is stopped in patients with BH4 responsiveness. Hence, BH4 could be used first as emergency treatment, independent of whether BH4 responsiveness can be proven.

Limitations of this study include that the setup was not prospective and that not all patients were tested for long-term BH4 responsiveness. Additionally, conclusions were largely based on 8-h neonatal BH4 loading tests due to treatment urgency, where 24-h tests would be more informative. Furthermore, the mean baseline Phe concentration was significantly higher in test I compared to test II. Another important remark is that test I and test II were performed with BH4 from different companies of which some of the first tests with 6R,S-BH4 which is less active. Notwithstanding the increase in positive neonatal BH4 loading tests (test I) in patients born after 1999, using the more active form of BH4 (6R-BH4), even in that patient group, a substantial number of patients had a negative test I but positive test II later on and confirmed long-term BH4 responsiveness.

## Conclusion

Although a positive neonatal 8-h BH4 loading test is predictive for long-term BH4 responsiveness, a negative 8-h or even a 24-h test can miss long-term BH4 responsive patients. As delays in treatment should be avoided, other alternatives to test for BH4 responsiveness at neonatal age, such as using genotype and starting BH4 treatment simultaneous with dietary treatment, should be investigated.

## Abbreviations

BH4: Tetrahydrobiopterin
PAH: Phenylalanine-4-hydroxylase
Phe: Phenylalanine
PKU: Phenylketonuria
